# Future Science OA 2019 early career researcher issue: foreword

**DOI:** 10.4155/fsoa-2019-0039

**Published:** 2019-05-03

**Authors:** Liam Michael Heaney, Joseph Martin

**Affiliations:** 1 School of Sport, Exercise & Health Sciences, Loughborough University, Loughborough, LE11 3TU, UK; 2 Future Science Group

LMH/JM: We would like to take this opportunity to welcome our readers to the *Future Science OA* (FSOA) Early Career Researcher (ECR) Issue. The concept of this issue was born from the understanding that the transition from doctoral studies to academic research can be daunting, with many challenges and hurdles in place. These challenges are not only limited to academic aspects (such as publishing manuscripts and acquiring research funding), but also cover general day-to-day motivation and individual well-being of the researcher. It is important that we understand these difficulties and actively look to support ECRs by providing advice through mentoring schemes and offering opportunities to demonstrate personal/academic capabilities wherever possible (e.g., conference attendance, networking, training courses). The availability of a rich and rewarding experience in the early postdoctoral years will actively encourage researchers to remain in academia (or pursue industrial-based research). This will, in turn, maintain the motivation and passion for research that leads to driving forward the science of the future and establishing personal feelings of self-efficacy and importance within our respective fields. The impact of these complex aspects of psychological health on ECRs cannot be underestimated and, therefore, we should all offer encouragement and recognition in helping promising individuals to flourish. For these reasons, this issue aims to provide advice and support to ECRs from their peers, as well as to offer a platform to publish excellent research that is being performed by developing scientists/researchers.

LMH: I am delighted to be involved in this year’s ECR issue. As a researcher who has been recently appointed onto academic tenure, I have experienced first-hand the obstacles and barriers that must be navigated in order to make the big leap into a career of academic research. One of the most difficult aspects of this transition is the realization and acknowledgment that rejection is a major part of our profession. This could be from the interview for that dream job, a submission to a leading journal or that pot of funding that is going to transform your career. While none of these situations is pleasant, we must learn to channel the disappointment to allow for ourselves to develop further and improve for the next opportunities that arise. It is important to remember that you are not the only one. Virtually, all ECRs will have experienced these difficulties, and many of the now internationally successful and leading researchers will have faced these challenges earlier in their career. It is vital that we all remain focussed and confident in our abilities by setting ambitious but realistic targets that can be regularly reassessed. By being conscious and aware that these difficult times will come, we are able to prepare correctly and react in a proactive manner to bring about the best possible outcomes. I would like to wish all ECRs who are reading this the best of luck for your future and encourage you to stay humble but never give up on your dreams.

JM: Here at *FSOA*, we have a number of initiatives to help support ECRs. It is important for us as a publisher to understand the needs and concerns of ECRs who are set to have a big impact in their field. In this special issue, we aimed to include a range of articles touching on what we believe are some of the most important topic areas for ECRs. The issue also features some exciting work led by ECRs.

We begin with a selection of interviews from our FS Early Career Research Award (ECRA) winner, Viviana Mucci (University of Antwerp), and finalists, Amy Winship (Monash Biomedicine Discovery Institute), Hannah Wardill (University of Adelaide and the University of Groningen) and Aya Mousa (Monash University). These interviews provide an insight into life as an ECR, what made them chose a career in their field, challenges and outlook to the future [1–4].

The ECRA, supported by FSOA, is an initiative that aims to recognize outstanding, highly talented and motivated researchers within the first 5 years of their first career position (in academia or industry). The winner receives membership to the FSOA early career advisory panel, a webinar to present their work and a monetary prize to support their career, among other things.

It is important to include topics that relate to many ECRs. Catherine Rawlins (Universite de Bordeaux) speaks about a very timely topic – mental health and how it can often be neglected in academia [5]. We would like to thank Catherine for writing this very honest and open editorial on such an important subject.

Kevin Deighton (Leeds Beckett University) and Javier Gonzalez (University of Bath) follow with an editorial based on the interesting concept ‘Academic Periodization’. Academic periodization is based on concepts used to optimize elite sporting performance, and this may represent an effective approach to help young academics reflect on their working practices in order to meet the demands of their roles [6].

Alistair Moss (University of Edinburgh) discusses both the challenge and importance of finding a mentor who can help to support the career of a young investigator in the editorial ‘See one, do one, teach one’ – finding your mentor in academic medicine’ [7].

In the first of our showcase pieces, guest editor Liam Heaney (Loughborough University) and his colleagues Owen Davies (Loughborough University) and Nicholas Selby (University of Nottingham) have described the potential use of short-chain fatty acids as therapeutic targets and/or biomarkers in renal disease patients in the editorial ‘Gut microbial metabolites as mediators of renal disease: do short-chain fatty acids offer some hope?’ [8].

As the winner of the ECRA, Viviana Mucci was awarded a free accelerated publication in FSOA. This research article on Mal de Debarquement syndrome, specifically aims to evaluate if symptoms change in patients with Mal de Debarquement syndrome during their pregnancy [9]. Viviana also had the opportunity to present her research on the same subject in a webinar organized as part of the ECRA.

A group of researchers based at the University of Sydney focus on the complex field of mass spectrometry tissue imaging. O’Rourke *et al.* provides a visual troubleshooting guide that will act as a reference point for a range of sample preparation mistakes and explanations for unusual or suboptimal data [10].

Heaney also authors the article ‘Probiotics: Current Landscape and Future Horizons’. This review discusses the recent rise in the global market for probiotic supplements, current research and drawbacks alongside the lack of translation from laboratory science to clinical application [11].

LMH/JM: We hope you enjoy reading the articles published in this ECR issue and invite you to contribute to discussion on this topic, and to future issues of *FSOA*.
